# The Best Medicines

**Published:** 1871-07

**Authors:** 


### THE BEST MEDICINES

In the world, more efficient in the cure of disease than all the potencies of the materia medica, are warmth, rest, cleanliness, and pure air. Some persons make it a virtue to brave disease, "to keep up" as long as they can move a foot or crook a finger, and it sometimes succeeds; but oftener the powers of life are thereby so completely exhausted that the system has lost all ability to recuperate, and slow and typhoid fevers set in, and carry the patient to a premature grave. Whenever walking or work is an effort, a warm bed and a cool room are the very first indispensable steps to a sure and speedy recovery. Instinct leads all beasts and birds to quietude and rest the very moment disease or wounds assail the system.

